# Prevalence, pattern and predictors of intimate partner violence amongst female undergraduates in Abia State, Nigeria; public health implications

**DOI:** 10.1186/s12905-024-03088-x

**Published:** 2024-04-25

**Authors:** Franklin Odini, Chidinma Amuzie, Kalu Ulu Kalu, Uche Nwamoh, Uloaku Emma-Ukaegbu, Michael Izuka, Uchechukwu Odini, Chukwubuike Ezepue

**Affiliations:** 1 Department of Community Medicine, Federal Medical Centre, Umuahia, Abia State Nigeria; 2 University of Nigeria Teaching Hospital, Ituku-Ozalla, Enugu State Nigeria

**Keywords:** Sexual abuse, Emotional abuse, Physical abuse, Nigeria, Students

## Abstract

**Background:**

Intimate Partner Violence (IPV) is the range of sexually, psychologically and physically coercive acts used against adult and adolescent women by a current or former male partner. It is a major public health problem globally. This study determined the prevalence, patterns and predictors of IPV amongst female undergraduates in Abia State.

**Methods:**

A cross-sectional study was conducted from January - February 2022 amongst 306 female undergraduates in Abia State. A mixed method of an online structured questionnaire created on Google forms & onsite self-administered questionnaire were deployed for data collection. Descriptive, bivariate and multivariate analyses were done using IBM SPSS Version 26.0. The level of significance was set at 5%.

**Results:**

A total of 306 respondents participated in the survey. The overall prevalence of IPV amongst female undergraduates was 51.2% (95% CI: 44.8-57.6%). Emotional abuse was the most common form of abuse 78.9%, followed by Physical abuse 42.0% and Sexual abuse 30.8%. Predictors of IPV reported include female earning/receiving more than their partner monthly (aOR = 2.30; 95% CI: 1.20–4.41); male (partner) alcohol consumption (aOR = 5.17; 95% CI: 2.46–10.88), being a smoker of cigarette/marijuana (aOR = 11.01; 95% CI: 1.26–96.25) and having witnessed domestic violence as a child (aOR = 3.55; 95% CI: I.56-8.07). Adverse effects such as unwanted pregnancies (12%), miscarriages (10%), eating/sleeping disorders (21%) and bruises (23%) amongst others were noted in some of the victims.

**Conclusion:**

Over half of all female undergraduates in Abia State have experienced IPV with emotional abuse being the commonest. Some Individual and relationship factors were identified as predictors of IPV. We recommend intensifying primary prevention campaigns against risk factors identified like smoking and alcohol consumption.

## Background

Intimate Partner Violence (IPV) is a major public health problem globally [1]. Alternate terminologies for it are domestic violence, family violence or relationship violence [1]. It is the commonest form of violence against women and one of the most pervasive human rights abuses against women [1]. Intimate Partner Violence refers to any behavior or act in the confines of an intimate relationship that results in physical, psychological or sexual harm to those in the relationship [2]. Repeated abuse in the same relationship is known as “Battering” [2]. The various forms of IPV include physical abuse, emotional or psychological abuse, sexual abuse and economic/financial abuse [2]. The 49th World Health Assembly declared that violence is a leading worldwide public health problem affecting people of all ages and sexes but especially women and children [3]. The worldwide prevalence of IPV is 35%, the prevalence in sub-Saharan Africa is reported to range from 20 to 71% and 38% of all murders of women worldwide are committed by intimate partners [4]. The 2013 Nigerian Demographic Health Survey (NDHS) reported that 25% of women in Nigeria reported having experienced IPV from their partner [5]. The consequences of IPV range from benign conditions such as bruises, cuts and so on to extreme consequences like suicidal thoughts, suicidal attempts and homicides [2]. A multi-country study stated that between 15 and 71% of female respondents reported to having experienced physical and/or sexual abuse by an intimate partner at some point in their life [6]. Amongst female undergraduates in Nigeria, a study done amongst female students in Ibadan, South-Western Nigeria reported an IPV prevalence of 44.1% amongst undergraduates [7], however, to the best of our literature search, there was no found study amongst female students in South Eastern part of Nigeria. Female students in tertiary institutions are a very important subset in the society as not only are they in/entering the most active reproductive stage of life, they also serve as an active pool for the workforce of the nation. Continuous unmitigated exposure to IPV will impact both the reproductive and economic fortunes of the nation amidst other negative impacts. IPV is very rampant but exhibits an iceberg phenomenon as the cases seen and reported are just a tip of the iceberg compared to the submerged portion of the iceberg which comprises of numerous unreported cases [5]. Women who are victims of the violence are exposed to a variety of untoward health outcomes [8]. The outcomes include low birth weight in women who were victims of IPV during pregnancy, unwanted pregnancy and induced abortion, sexually transmitted infections (STIs), physical injury, temporary or permanent disability, depression, alcohol abuse, post-traumatic stress syndrome [8]. In extreme cases, IPV has been seen to lead to homicides and suicides [8]. The 2013 NDHS reported that amongst women who experienced physical violence in the 12 months preceding study, 33% reported varying degrees of physical injuries [5]. In addition to the above, amongst female students, IPV can also lead to inability to concentrate on their studies, absenteeism, interrupted studies and so on [7]. One of the recommendations from the 49th World Health Assembly was that member states assess the burden of the problem of IPV in their territories and initiate public health activities to address this problem [3]. In addition, WHO has posited that though addressing the problem of IPV is through primary prevention, the very first step of this process is through determination of prevalence in member states and building of the knowledge bank concerning IPV. In Nigeria, especially in the South Eastern part where this study was carried out, there is paucity of information among the young population and those in dating/courtship. There is also a need to assess this burden among the undergraduates, because there is a greater risk attached to this specified population. Alcohol abuse, substance abuse, multiple partners and so on are some of the factors associated with IPV from previous studies that are rife in University settings, hence the appropriateness of this setting for this study. With the prevalence data and the developed knowledge bank, relevant stakeholders can be objectively engaged with facts and data, and the problem of IPV can be holistically addressed. This study will aid in addressing IPV by taking the critical first step of building the knowledge bank and providing data on the burden, determinants and consequences of IPV amongst female undergraduates in Abia State. This study aimed to determine the prevalence, patterns and predictors of IPV amongst female Undergraduates in Abia State.

## Methods

### Study setting and design

This was a Cross-sectional study conducted in Abia State. Abia state is one of the five states in the South-East zone of Nigeria. It has an estimated population of 3,784,355 in 2017 projected from the 2006 national population census based on an annual growth rate of 3.0% [9]. Micheal Okpara University of Agriculture Umudike (MOUAU) and Abia State University (ABSU) were used for this study. MOUAU is located in Umudike, Abia State and the most recent statistics indicates the school has a student’s population of 27,750 undergraduates with over half being females [10]. ABSU recent statistics show the school has a student’s population of about 20,900 undergraduates with over half being females [11].

### Study population

Female undergraduates in MOUAU and ABSU Abia State made up the study population. This population was irrespective of factors like age, year of study, marital status, ethnicity, marital status and denomination. The inclusion criteria included ABSU and MOUAU female students aged ≥ 18 years who were in a current relationship or had ever been in a relationship in the last 1 year. Eligible participants who were ill or pregnant or did not consent were excluded from the study.

### Sampling technique

A five stage multi-stage sampling was used to select participants. In the first stage, using Simple random sampling (balloting method), 2 Universities were selected from the 6 Universities in Abia State. Thereafter, using Simple random sampling (balloting method), Faculty of Law and Faculty of Medicine and Health Sciences were selected from ABSU while Faculty of Management Science and Faculty of Veterinary Medicine were selected from MOUAU. In the third Stage, using Simple random sampling (balloting method), Department of Clinical Medicine and Department of Law was selected from ABSU while Accounting Department and Veterinary Medicine Department was selected from MOUAU. In the Fourth stage, each selected Department was stratified according to their respective years of study and proportionate to size sampling was done to get the required sample size in each stratum.

Finally, using each Department’s student register as the sampling frame, Simple random sampling was done by generating random numbers using Open Epi and these were used to select the actual respondents from the register. All consenting selected students were given the questionnaires by the research assistant who were students drawn from each selected class. Contacts of selected respondents were obtained from the Class Department reps or Class Whatsapp Group and a total of 146 respondents had the links sent to their Whatsapp messengers after confirming their identity over a phone call. A total of 160 selected respondents who were not on the Whatsapp groups and did not have internet enabled phones to receive the questionnaire link were visited either in class or their hostels and issued the hardcopy questionnaires by the Research Assistants. The data from the hardcopy questionnaires were collated thereafter with an Excel sheet.

### Sample size determination

Using the proportion of 23.6% who had experienced IPV from a previous study amongst females in Osogbo Nigeria [12], and precision of 5%, the sample size was calculated using the single proportion population formula. It is given as n = (Zα)^2^pq/d^2^.

Zα = 1.96, *p* = 0.236, q = 0.764 and d^2^ = 0.0025.

(1.96)^2^(0.236 × 0.764)/0.0025.

The minimum sample size was estimated to be 277. However, providing for a non-response rate (r) of 10% (0.1), the sample size was estimated by the formula n/1-*r* = 277/0.9 = 307.

Hence, the minimum sample size was 307.

### Study tool and data collection process

Rigorous literature review was done and the structure of the questionnaire was formed using published research. The questionnaire was modified from the 2013 NDHS survey on IPV in Nigeria [5] and consisted of 4 sections labelled A-D. With the help of Google forms, a cloud-based survey tool powered by Google, a semi-structured questionnaire was created for data collection over a 2-month period (January - February 2022). Hardcopy questionnaires were also printed and given to respondents who did not have access to smart devices or functional internet. The questionnaire was validated using the face and content validity techniques. The introductory segment of the questionnaire emphasized the privacy and confidentiality of the respondents’ responses. The pretest was done amongst students of one of the other Universities not selected for this study. This helped to improve the diction and appropriateness of the questionnaire. The questionnaire had different sections. **Section A** included information on the Socio-demographics such as age, level of study, religion, denomination, HIV status of partner, occupation of partner, partners level of education and so on. **Section B** obtained information on occurrence of IPV and pattern. It contained questions such as;

- Does (did) your (last) husband/partner ever: (a) Say or do something to humiliate you in front of others? (b) Threaten to hurt or harm you or someone close to you? (c) Insult you or make you feel bad about yourself?
- Does (did) your (last) husband/partner ever do any of the following things to you? (d) Push you, shake you, or throw something at you? (e) Slap you? (f) Twist your arm or pull your hair? (g) Punch you with his fist or with something that could hurt you? (h) Kick you, drag you, or beat you up? (i) Try to choke you or burn you on purpose? (j) Threaten or attack you with a knife, gun, or any other weapon? (k) Physically force you to have sexual intercourse with him even when you did not want to? (l) Physically force you to perform any other sexual acts you did not want to? (m) Force you with threats or in any other way to perform sexual acts you did not want to?
- Does (did) your (last) husband or partner ever do any of the following things to you? (n) Refuse to give you money for your basic needs.

Answering Yes to one or more of the items in (a) to (c) above constituted evidence of emotional violence, answering Yes to one or more of items in (d) to (j) constituted evidence of physical violence and answering Yes answer to one or more of items in (k) to (m) constituted evidence of sexual violence.

**Section C** obtained information on determinants of IPV such as partners alcohol consumption, monthly allowances/earnings of couple, partners cigarette or marijuana use, type of relationship (open, committed, single or married), partner having concurrent relationships outside marriage, witnessing or experiencing domestic violence as a child, and presence of aggressive behavior in spouse. **Section D** sought information on the health consequences of IPV on the abused such as presence or absence of bruises, unwanted pregnancies, STDs, injuries, suicidal thoughts and so on.

The questionnaire link was sent to the selected students via their Whatsapp Messenger after confirming their identity via a phone call by the Research Assistant. Research assistants also distributed hardcopies of the questionnaire to eligible participants who were unable to fill the online form for a myriad of reasons. A total of 306 responses was received (153 from each university) and consisted of 160 responses from the online link and 146 responses from the physical questionnaires (Fourteen eligible respondents withdrew consent midway into data collection). A total of 14 Research assistants selected from each class of every Department selected for this study. They were trained for 2 days by 3 of the Researchers on how to apply the questionnaire, how to transcribe the completed hardcopy forms into an Excel sheet already provided, how to provide help to the respondents during the completion process and escalate difficult problems to the researchers if any.

### Measurement of variables

The primary outcome measure was IPV amongst the female undergraduates sampled. Answering Yes to any of the questions in Section B of the Questionnaire were scored 1, No was scored 0. Answering yes to any of the questions in (a) – (c), (d) – (j) or (k) – (m) of Section B of the questionnaire confirmed emotional, physical or sexual abuse respectively. Other independent variables measured included Socio-demographic factors, Determinants of IPV and outcomes of IPV in victims.

### Statistical analysis

Data coding, entry, cleaning, and analysis were done using IBM SPSS version 26 statistical program for Windows. Descriptive statistics was used to characterize the sample and study variables. Associations between independent variables and IPV were assessed with crosstabulations. Bivariate logistic regression model was used to identify associated factors of IPV in the study population and multivariate logistic regression model was used to determine the significant independent predictors of IPV. The level of significance was predetermined at a *p*-value of < 0.05.

### Ethical approval and consent to participate

Study was approved by the **Health Research Ethics Committee** of Federal Medical Centre Umuahia, Nigeria. Informed Consent was obtained from the respondents prior to filling the questionnaire. All methods in this study were carried out in accordance with the Declaration of Helsinki.

## Results

A total of 306 participants participated in the study out of 320 eligible respondents approached. The mean age was 24.02 ± 5.77 years. Those aged 20-24years constituted the majority of the respondents (45.8%). Majority of the respondents were Christians (90.2%) Among the respondents, the majority (34.6%) were in year three of study. 65% of the respondents were in one form of relationship or the other. A greater proportion of the respondents were currently in the category of ‘in a relationship/ had been in a relationship’ (81.7%). (Table 1)

Out of the 250 respondents who were currently in a relationship or ever been in a relationship, majority of their partners were undergraduates (48.4%) and were employed (72.8%). A greater proportion of their partners were HIV negative 209 (83.6%) (Table 2).

**Fig. 1:**
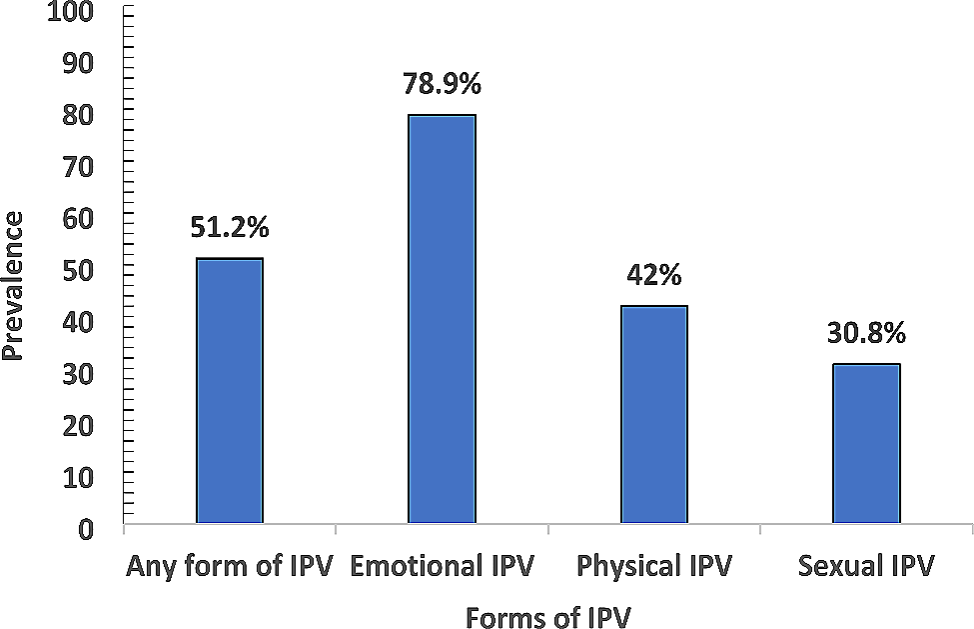
Prevalence and forms of IPV amongst female undergraduate students in Abia state

The prevalence of IPV amongst female undergraduates was 51.2% and the most prevalent form of IPV suffered by these undergraduates was Emotional abuse (78.9%). (Fig. 1).

Consequence of IPV reported in this study varied from Physical, Sexual and Emotional consequences, with the commonest being bruises (29.9%), fractures and abdominal injuries (18.2%), unwanted pregnancies (9.4%), eating or sleeping disorders (16.4%), low self-esteem and anxiety (13.3%) as well as panic attacks (11.7%). Attempted suicide was low amongst them (4.1%). (Table 3).

**Fig. 2:**
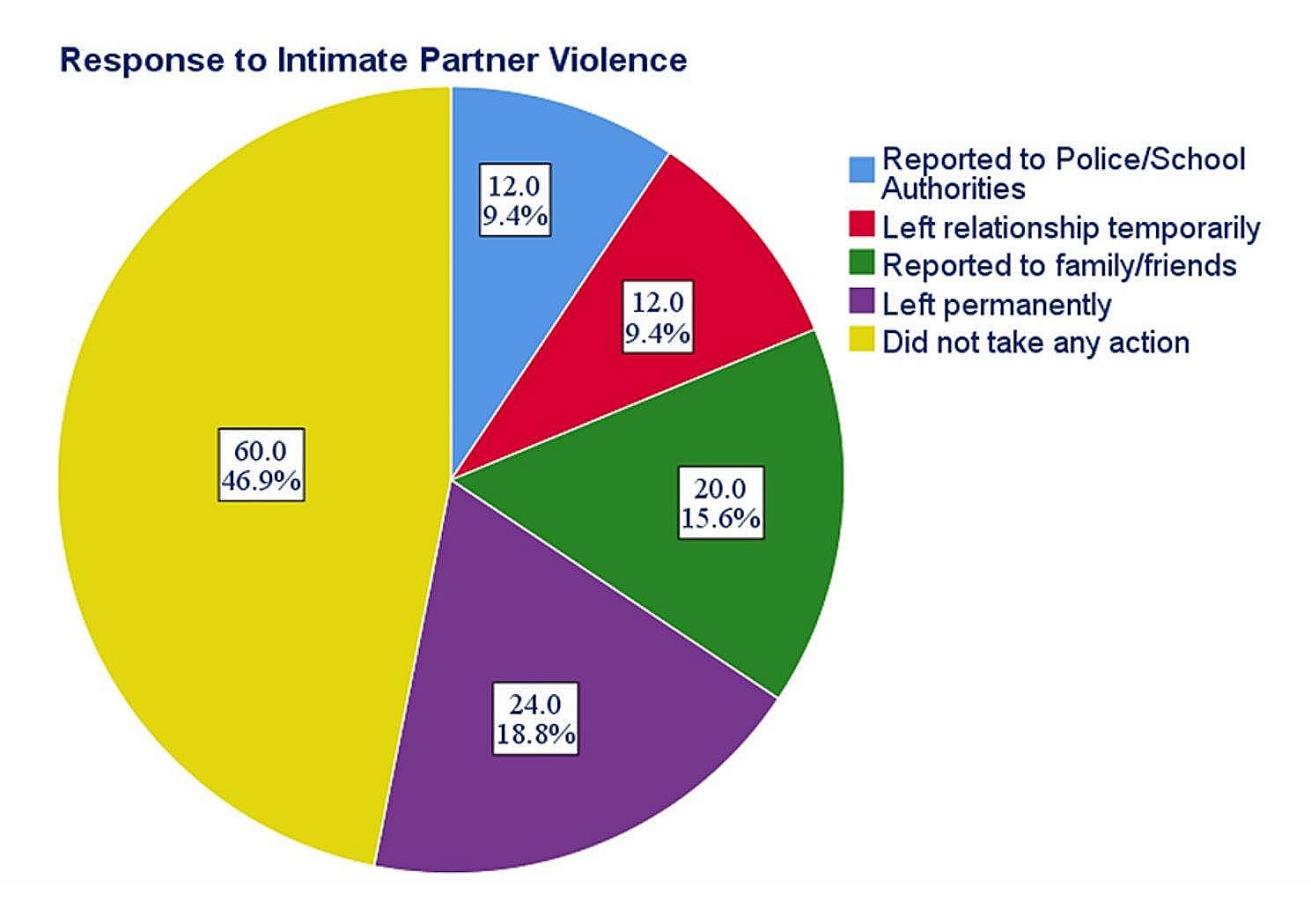
Action taken by female undergraduate students in Abia state following IPV

Most of the respondents who had suffered IPV, did not take any action (46.9%), while a little proportion of victims reported to the police/school authorities (9.4%). (Fig. 2).

**Table 1:** Socio-demographic and sexual characteristics of respondents (*N* = 306)

Variables	Frequency	Percentage (%)
**Age (years)**		
< 20	58	19.0
20–24	140	45.8
25–29	62	20.3
≥ 30	46	15.0
Mean ± 2SD	24.02 ± 5.77	
**Religion**		
Christianity	276	90.2
Islam	23	7.5
Traditionalist	7	2.3
**Level of study**		
100	46	15.0
200	65	21.2
300	106	34.6
400	69	22.5
500	14	4.6
600	6	2.0
**Relationship status**		
Single	107	35.0
Married	31	10.1
Committed	116	37.9
Open	52	17.0
**Ever had a partner**		
Yes	250	81.7
No	56	18.3
**Age at first sexual intercourse**		
< 18 years ≥ 18 years No sexual intercourse yet	59 155 92	19.3 50.7 30.1
**Conditions of first sexual intercourse**		
Consented Coerced/forced No intercourse yet	182 32 92	59.5 10.5 30.1
**HIV status of respondent**		
Negative Positive Unknown	261 8 37	85.3 2.6 12.1

**Table 2:** Socio-demographic characteristics of respondents’ partners

Variables	Frequency	Percentage (%)
**Educational Status**		
Secondary	6	2.4
Undergraduate	121	48.4
Postgraduate	95	38.0
Others	28	11.2
**Occupation**		
Employed	182	72.8
Not Employed	68	27.2
**HIV status**		
Negative	209	83.6
Positive	9	3.6
Unknown	32	12.8
**Does your partner smoke? (cigarette/marijuana)**		
Yes	11	4.4
No	239	95.6
**Does your partner take alcohol?**		
Yes	41	16.4
No	209	83.6

**Table 3:** Consequences of intimate partner violence amongst female undergraduate students in Abia state

Consequences of IPV	N (%)
**Physical consequence**	
Bruises	23 (29.9)
Fractures	14 (18.2)
Chest injuries	14 (18.2)
Abdominal injuries	13 (16.9)
Lacerations and open wounds	13 (16.9)
**Sexual consequence**	
Pregnancy complications	7 (5.5)
Miscarriages	10 (7.8)
Unsafe abortions	8 (6.3)
STIs	9 (7.0)
Unwanted pregnancies	12 (9.4)
**Emotional consequence**	
Alcohol or drug abuse	11 (8.6)
Feeling of shame	16 (12.5)
Panic attacks	15 (11.7)
Eating/sleeping disorders	21 (16.4)
Low self esteem	17 (13.3)
Anxiety	17 (13.3)
Suicidal thoughts	11 (8.6)
Attempted Suicide	4 (3.1)

**Table 4:** Associated factors and significant predictors of intimate partner violence (IPV) among female undergraduate students in Abia state

Variables	IPV		COR (95% CI)	*P* value	aOR (95% CI)	*P* value
	Yes(%)	No(%)				
**Do you earn/receive more than your partner per month**						
Yes	50(65.8)	26(34.2)	2.37(1.35–4.14)	0.002	2.30 (1.20–4.41)	0.012
No	78(44.8)	96(55.2)				
**Does your partner consume alcohol**						
Yes	54(78.3)	15(21.7)	5.21(2.73–9.91)	0.001	5.17 (2.46–10.85)	0.001
No	74(40.9)	107(59.1)				
**Do you consume alcohol**						
Yes	36(69.2)	16(30.8)	2.59(1.35–4.97)	0.003	0.98 (0.43–2.22)	0.954
No	92(46.5)	106(53.5)				
**Does your partner smoke cigarette/marijuana**						
Yes	29(76.3)	9(23.7)	3.68(1.66–8.14)	0.001	1.43 (0.55–3.70)	0.466
No	99(46.7)	113(53.3)				
**Do you smoke cigarette/marijuana**						
Yes	10(90.9)	1(9.1)	10.25(1.29–81.36)	0.007	11.01(1.26–96.25)	0.030
No	118(49.4)	121(50.6)				
**Does your partner have a concurrent relationship**						
Yes	15(65.2)	8(34.8)	1.89(0.77–4.64)	0.158	-	-
No	113(49.8)	114(50.2)				
**Ever witnessed domestic violence as a child**						
Yes	30(73.2)	11(26.8)	2.57(1.21–5.47)	0.002	3.55 (1.56–8.07)	0.002
No	98(46.9)	111(53.1)				
**Is your partner regularly involved in fights with other men or participate in riots**						
Yes	13(68.4)	6(31.6)	2.18(0.80–5.94)	0.118	-	-
No	115(49.8)	116(50.2)				

In the Bivariate logistic regression model, monthly allowance/income, alcohol consumption, smoking and having witnessed domestic violence as a child were significantly associated with suffering IPV. Undergraduate females who earned/receive more than their male partners were more than two folds likely to suffer IPV (OR = 2.37; 95% CI: 1.35–4.14). Those who consume alcohol (OR = 2.59; 95% CI: 1.35–4.97) and those whose partners consume alcohol (OR = 5.21; 95% CI: 2.73–9.91) were more likely to suffer IPV compared to their counterparts. Similarly, female undergraduates who smoke (OR = 10.25; 95% CI: 1.29–81.36) or who have partners’ that smoke (OR = 3.68; 95% CI: 1.66–8.14) were also more likely to suffer IPV compared to their counterparts. Respondents who witnessed domestic violence as children were about three-folds more likely to suffer IPV compared to those who never witnessed domestic violence as children (OR = 2.57; 95% CI: 1.21–5.47).

After controlling for other variables in the multivariate logistic regression model, the predictors of Intimate Partner Violence were: female earning/receiving more than male partner monthly (aOR = 2.30; 95% CI: 1.20–4.41); male (partner) alcohol consumption (aOR = 5.17; 95% CI: 2.46–10.88), female smoker of cigarette/marijuana (aOR = 11.01; 95% CI: 1.26–96.25) and having witnessed domestic violence as a child (aOR = 3.55; 95% CI: I.56-8.07) (Table 4).

## Discussion

IPV was quite rampant amongst female undergraduates in this study, with a prevalence rate of (51.2%). This suggests that more than half of Female Undergraduates in Abia State have been abused by their partners with its attendant consequences on their emotional, physical and sexual health. This is higher than that reported in a similar study conducted in Ibadan Nigeria amongst female students [7]. A similar study conducted amongst undergraduate students in Northern Nigeria by Iliyasu et al. [13] showed a higher prevalence of IPV at 58.8% among females. In addition, student nurses were studied in Abakaliki, southeastern part of Nigeria and the prevalence of IPV although reported to be lower than our findings (48.7%) was still high amongst the respondents [14]. This calls for a need for a thorough history and examination of female undergraduates presenting to the hospital with unexplained injuries or conditions. In addition, another study from Africa, Zambia to be precise, reported a prevalence of 53.5% which is very close to the prevalence reported in this study [15] This suggests that IPV is not peculiar to Nigeria alone and some common characteristics may exist between countries in the same continent to explain this occurrence. There is need for campaigns to mitigate this burning issue amongst African females both at the individual and at higher level. The prevalence reported in this study is higher than what was reported in Spain (19.2%) [16]. The lower prevalence rates of IPV in Europe compared to this study and the above reviewed studies in Africa may also be due to better law enforcement against IPV perpetrators serving as a deterrent to others in more advanced countries of Europe.

Our study revealed that forms of IPV were 78.9%, 42.0% and 30.8% for emotional, physical and sexual violence respectively amongst our respondents. This corroborates with the study done at Ibadan [7] and is slightly similar in pattern to what was reported in the NDHS 2013 study for Abia State differing only in which form was the commonest [5]. From the 2013 NDHS, a prevalence of 12.9%, 21.2% and 5.5% for emotional, physical and sexual abuse respectively were reported in Abia State [5]. Both studies agreed that the least common form of abuse amongst women in Abia State is sexual abuse but differed in findings for the commonest form. Our study was restricted to female undergraduates in Abia State while the NDHS surveyed all women in Abia state and this may account for the disparity in prevalence and commonest patterns reported. The pattern of IPV reported in this study is also very similar to the patterns reported in other studies from other parts of Nigeria [7, 12, 13, 17–19]. It is seen that studies from other parts of Africa showed a different pattern of IPV occurrence amongst women [6, 20]. To be more elaborate, studies from Ethiopia, Namibia and Tanzania all reported the prevalence of physical, sexual and emotional violence as 48.7%, 58.6% and 9.2% respectively, 30.6%, 16.5% and 8.4% respectively and 46.7%, 30.7% and 15.3% respectively [6]. This pattern reported in these countries suggest that emotional violence is the least common form of IPV amongst the three studied forms of IPV which is different from what was reported in this study. Cultural differences, different levels of patriarchy and legalization of male roles across the different countries and regions in Africa may be responsible for this deviation in pattern.

In Europe, studies from Spain showed a similar pattern of IPV to that reported in this study [16]. While this study was not on students, they had comparable age incidence with our respondents. The Public health significance of increased forms of IPV amongst female undergraduates are vast and range from non-adherence to medications, poor academic performance, reduced health seeking actions, increased associated morbidities and so on and all these may increase mortality and reduce productivity in the society [21]. We reported a wide range of the consequences of IPV, with the commonest being bruises (29.9%), fractures and chest injuries (16.4%), unwanted pregnancies(9.4%), eating/sleeping disorders (16.4%), feeling of shame (12.5%), low self-esteem and anxiety (13.3%) as well as suicidal thoughts (8.6%). This is in keeping with reports from other studies [22–24]. 

Individual factors are factors peculiar to the individual and they have been found to be risk factors for IPV [2, 25]. This study reported that females who witnessed domestic violence as children were over 3 times more likely to experience IPV later in life. This is congruent with the finding from Ibadan Nigeria where it was reported that students who had exposure of inter-parental violence were significantly associated with experiencing IPV later in life [7]. Witnessing abuse as children especially amongst parents may have a profound normalization effect on the child. Future abuse may not be seen for what it is due to the numbing effect that her childhood experience has had on her psyche thus making her more vulnerable to abuse [26]. This mindset may also be transferred down to their children thereby resulting in inter-generational normalization of abuse and various forms of violence [26]. 

Partner frequent use of alcohol is another individual factor associated with IPV amongst female undergraduates in this study where it was reported that females whose partners use alcohol frequently were at least five times at risk of experiencing IPV. The disinhibition associated with alcohol use may result in heightened response of the victim’s partner to minimal provocation from the victim. Frequent alcohol use by partner may also be his adaptation mechanism to the numerous societal and/or economic challenges facing the nation, for example owed salaries, downturn in business fortunes, academic problems and so on. The 2013 NDHS corroborated this finding as it reported that women whose husbands or partners get drunk often were more likely to report IPV than women whose husbands drink minimally or don’t drink at all [5]. This is also in keeping with findings from other related studies from [7, 13, 19, 27], . Female smokers of cigarette or marijuana were also reported in this study to be eleven times at risk of experiencing IPV. This is very similar to what was reported in a study done in Ibadan Nigeria amongst female students where it was reported that there was a higher rate of IPV amongst students who were smokers than amongst those who were not smokers [7]. This was also supported by other studies carried out on young adults in the United States [28, 29]. Cigarette and marijuana use can influence the occurrence of IPV the same way alcohol does. Both agents lead to dependence and disinhibition which may alter the partners sensorium and lead to violent responses to provocation, responses which may not have occurred had the substances not been used.

We reported that female undergraduates who received/earned more than their partners monthly were at least twice at risk of experiencing IPV. Earning more than one’s partner in a patriarchal/male dominated society may lead to IPV as the men may not be comfortable with knowing their partners earn more than they do and are financially superior. This may lead to inferiority complex problems on the man’s part and tension build-up, which may eventually lead to violence. This finding was congruent with other studies [30, 31].

Contrary to our findings, Iliyasu et al. [8] reported that religious affiliation, ethnicity, indigeneship, marital status, campus residence and faculty affiliation were significant predictors of GBV. This further shows the impact of religion as well as environmental factors on IPV. Interventions geared towards reducing knowledge of IPV in females has been shown to be impactful [32]. It is therefore essential that such interventions be incorporated into the guidance and counselling units in the tertiary institutions.

In conclusion, IPV was prevalent amongst over half of female undergraduates in Abia State, with, emotional abuse being the commonest form of abuse while sexual abuse was the least common. Female undergraduate earning/receiving more than male partner monthly, male (partner) alcohol consumption, female smoker and having witnessed domestic violence as a child were all significant predictors of IPV amongst female undergraduates.

We therefore recommend that primary and secondary prevention interventions targeted at curbing the identified risk factors of IPV be instituted for both young men and women in Abia state tertiary institutions. Additionally, there is need for institutions to implement adequate reporting and effective response mechanisms to assist victims and mitigate the myriad of negative outcomes.

The major strength of this study is that it was among the first studies in South Eastern region of Nigeria to explore IPV holistically amongst female undergraduates to the best of our knowledge. Secondly, having used two major tertiary institutions within the state, we got a good representative spread of undergraduate respondents which validates generalization of the study. Finally, both online and physical questionnaires were used to reduce the possibility of selection bias and restricting respondents to only those with access to smart devices (phones and tablets) connected to the internet.

Limitations of this study is that there may not have been full disclosure from the respondents, considering the fact that IPV is a very sensitive issue. Some people may be guarded about providing information on abuse and some may have recall bias of some events or not want to talk about it at all. Also, due to the cross-sectional design of this study, only associations of IPV could be tested for and this study was unable to determine causal relationships or describe temporal associations between some of the factors associated with experience of violence. Some of the above limitations were mitigated by assuring respondents of full confidentiality of their responses, advising them to fill the questionnaire (either online or physical) in a private space with little distractions, advising them in the introductory section of the questionnaire not to proceed if some of the questions may cause them any form of distress and providing the researchers’ contact for easy accessibility incase the respondent needed any form of psychosocial support or help with reporting recent or ongoing abuse.

## Data Availability

The dataset analyzed in the study are not publicly available due to concern for misuse and breach of privacy/confidentiality but are available from the corresponding author on reasonable request.

## Abbreviations

ABSU: Abia State University
AIDS: Acquired Immune Deficiency Syndrome
aOR: Adjusted Odds Ratio
CI: Confidence Interval
CDC: Center for Disease Control
FMC: Federal Medical Centre
HIV: Human Immunodeficiency Virus
IPV: Intimate Partner Violence
MOUAU: Micheal Okpara University of Agriculture Umudike
NDHS: Nigerian Demographic and Health Survey
SPSS: Statistical Package for Social Sciences
STIs: Sexually Transmitted Infections
UN: United Nations
WHO: World Health Organisation
WLWHA: Women Living With HIV/AIDS
